# Data on production of mammalian stable cells expressing secretory BEFV transmembrane deleted G protein

**DOI:** 10.1016/j.dib.2019.104603

**Published:** 2019-10-03

**Authors:** Payuda Hansoongnern, Challika Kaewborisuth, Porntippa Lekcharoensuk

**Affiliations:** a Interdisciplinary Graduate Program in Genetic Engineering, The Graduate School, Kasetsart University, Bangkok, 10900, Thailand; b Center for Advanced Studies in Agriculture and Food (CASAF), KU Institute of Advanced Studies, Kasetsart University, Bangkok, 10900, Thailand; c Virology and Cell Technology Laboratory, National Center for Genetic Engineering and Biotechnology, National Science and Technology Development Agency, Pathum Thani, 12120, Thailand; d Department of Microbiology and Immunology, Faculty of Veterinary Medicine, Kasetsart University, Bangkok, 10900, Thailand

**Keywords:** Bovine ephemeral fever virus, Transmembrane deleted G protein, Mammalian stable cell, Protein expression

## Abstract

Generation of stable cell lines is a widely used technique for continuous recombinant protein production. Advantages of the constitutive stable over the transient protein expression are uniformity of the expression across cell populations as well as high quantity and consistency of the protein yields. This data describe step-by-step procedure for the production of glycoprotein without a transmembrane domain (GΔTM) of bovine ephemeral fever virus (BEFV) by mammalian stable cells. LentiX-293T cells were transfected with four plasmid constructs to generate a recombinant lentivirus. Subsequently, 293T cells were transduced by the recombinant virus and the polyclonal stable cell pools were then selected by puromycin. Next, limiting dilution was performed from each cell pool to isolate the monoclonal stable cells expressing GΔTM protein. Western blot analysis showed that all monoclonal cell clones could stably express GΔTM protein. The data confirms that the stable 293T cell line expressing the secretory GΔTM protein is an attractive platform for antigen production.

**Table:** Specifications Table

Subject area	Immunology and Microbiology
More specific subject area	Applied Microbiology and biotechnology
Type of data	Figures and Tables
How data was acquired	Western blot analysis
Data format	Raw data
Experimental factors	Western blot was performed for GΔTM expression using anti-his tag and anti-mouse IgG HRP as a primary and secondary antibodies, respectively.
Experimental features	LentiX-293T cells were transfected with pLVX_GΔTM vector along with other vectors to generate a recombinant lentivirus. Then, 293T cells were transduced with the recombinant virus and positive clones expressing GΔTM protein were further selected by antibiotic selection.
Data source location	Bangkok, Thailand
Data accessibility	Data are within this article
Related research article	P. Hansoongnern, C. Kaewborisuth, K. Wasanasuk, P. Chankeeree, S. Poonsuk, C. Lekcharoensuk, P. Lekcharoensuk, The immunogenicity of the secretory GΔTM protein of bovine ephemeral fever virus stably expressed by mammalian cells, Vet. Microbiol. 233 (2019) 113–117 [1].

**Table:** 

**Value of the Data** • The data provide complete details to generate recombinant lentivirus expressing BEFV GΔTM protein. • The data provide step-by-step procedure for generating mammalian stable cells secreting BEFV GΔTM protein. • The data can help researchers to effectively produce an interest protein from mammalian stable cells.

## 1 Data

**Fig. 1:**
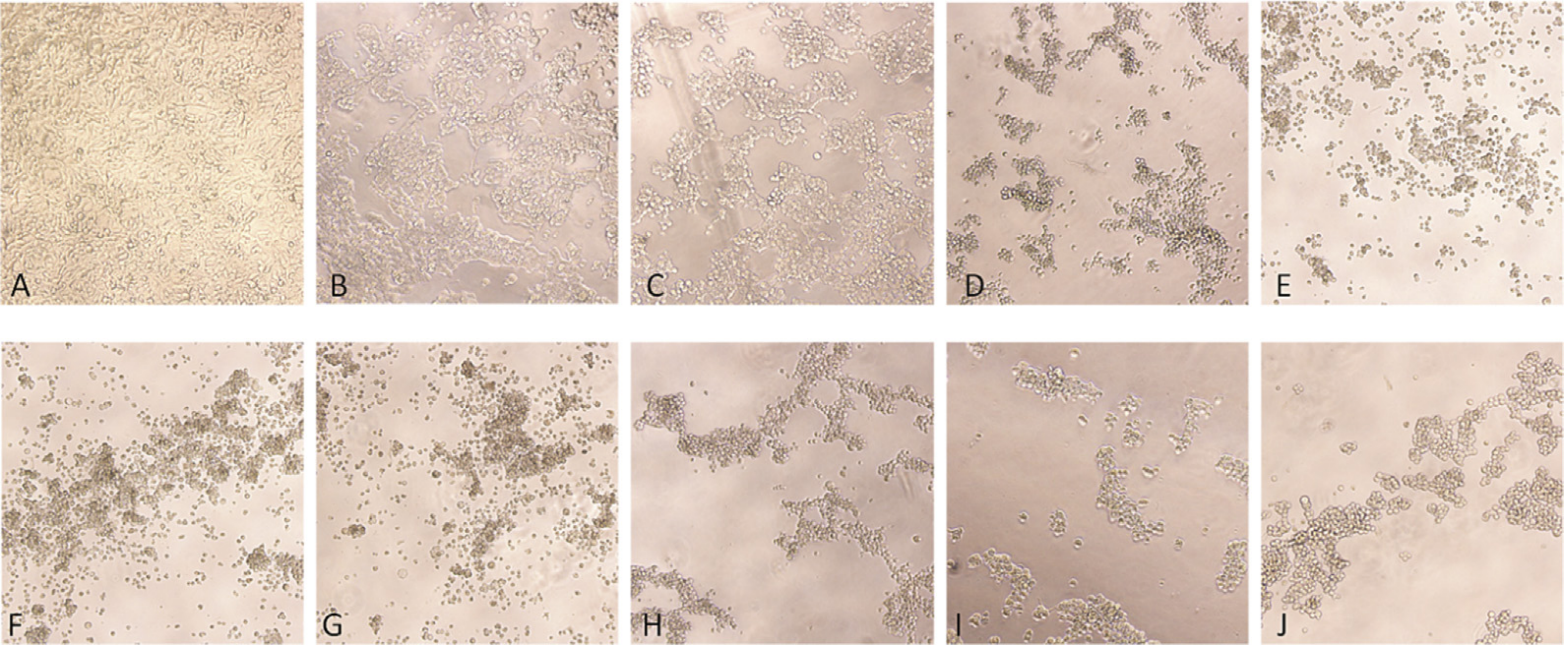
293T cells cultured in the medium containing various concentrations of puromycin in order to determine the minimum concentration of the drug that could completely killed all cells within 3–5 days. (A) 293T cells without puromycin. (B–J) 293T cells treated with puromycin at 0.5, 1, 3, 5, 8, 10, 25, 50, and 100 μg/ml, respectively. All 293T cells cultured in the selecting medium containing 3 μg/ml puromycin died at day 4 post antibiotic treatment (D) whereas the cells treated with the higher concentration of puromycin died within 2 days (E–J).

**Fig. 2:**
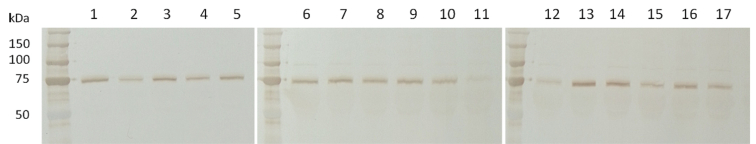
Reactivity between GΔTM protein from culture medium supernatant and anti-histidine tag monoclonal antibody as examined by Western blot analysis. Lanes 1–17 indicate GΔTM protein secreted from 293T_GΔTM stable cell clone no.1–17, respectively.

Generation of mammalian stable cells expressing BEFV GΔTM protein in secretory form resulted in constitutive expression of the target protein [1]. To produce the stable BEFV GΔTM secreting cells, recombinant lentiviral particles were generated and rescued by co-transfection of LentiX-293T cells with four plasmid constructs followed by determination of lentivirus titration. The lentivirus RNA copy number acquired from our experiment corresponded to a raw copy number of 1.2 × 10^7^ copies by the qRT-PCR standard curve which equal to 4 × 10^9^ copies/ml. This is typically 100-fold lower than normally observed in the infectious titer as measured by transduction units (TU/mL) [2]. Results of standard curve optimization and viral RNA copy number were shown in Table 1, Table 2, respectively. Subsequently, transduction of 293T cells with the recombinant lentivirus was performed and the transduced cells were selected by puromycin resistant characteristics. The optimal concentration of puromycin for the selection process was determined and it was 3 μg/ml (Fig. 1). Monoclonal stable cells secreting BEFV GΔTM were isolated from antibiotic resistant cell pools by the limiting dilution technique. All monoclonal stable cell clones stably expressing secretory BEFV GΔTM protein as examined by Western blot analysis (Fig. 2).

**Table 1:** Standard curve optimization for lentivirus RNA copy number quantification.

RNA concentrations (ng/ul)	Total RNA (ng)	Copy numbers (2 × 10^x^)	Cq
50	100	11	3.52
50	100	11	3.52
5	10	10	8.17
5	10	10	8.28
0.5	1	9	12.21
0.5	1	9	12.04
0.05	0.1	8	16.27
0.05	0.1	8	16.27
0.005	0.01	7	19.65
0.005	0.01	7	19.72
0.0005	0.001	6	23.58
0.0005	0.001	6	23.54
0.00005	0.0001	5	27.22
0.00005	0.0001	5	27.15
0.000005	0.00001	4	30.62
0.000005	0.00001	4	30.53

**Table 2:** Quantitative real time RT-PCR of viral RNA copy number from lentivirus sample.

Sample	Dilutions	Ct value	Copy number
Std-1	10^11^	5.13	200000000000.00000
Std-1	10^11^	5.49	200000000000.00000
Std-2	10^10^	8.94	20000000000.00000
Std-2	10^10^	8.52	20000000000.00000
Std-3	10^9^	12.35	2000000000.00000
Std-3	10^9^	12.56	2000000000.00000
Std-4	10^8^	16.37	200000000.00000
Std-4	10^8^	16.14	200000000.00000
Std-5	10^7^	20.31	20000000.00000
Std-5	10^7^	20.55	20000000.00000
Std-6	10^6^	24.08	2000000.00000
Std-6	10^6^	24.12	2000000.00000
Std-7	10^5^	27.83	200000.00000
Std-7	10^5^	27.33	200000.00000
Std-8	10^4^	29.66	20000.00000
Std-8	10^4^	29.56	20000.00000
Sample-1	10^−1^	24.12	1326041.51081
Sample-1	10^−1^	24.28	1198896.77120
Sample-2	10^−2^	28.02	110807.70660
Sample-2	10^−2^	28.01	111306.77372
Sample-3	10^−3^	31.77	10097.38460
Sample-3	10^−3^	31.51	11931.16565
Sample-4	10^−4^	ND	
Sample-4	10^−4^	ND	
Sample-5	10^−5^	ND	
Sample-5	10^−5^	ND	

Std = standard GagP24 RNA copy number. ND is referred to no detection.

## 2 Experimental design, materials and methods

### 2.1 Production of recombinant lentivirus

pLVX_GΔTM construct used for transfection consisted of N-terminal BiP secretory signal sequence followed by GΔTM in which signal peptide, transmembrane domain, and cytoplasmic tail were deleted. To generate recombinant lentiviruses, LentiX-293T cells were seeded at 4.5 × 10^6^ cells in a 10 cm^2^ plate and incubated overnight at 37 °C with 5% CO_2_. The LentiX-293T cells were co-transfected with a transfer vector containing GΔTM (pLVX_GΔTM) produced in our laboratory [1] and three helper plasmids including pMDLg/pRRE, pRSV-Rev, and pMD2.G [3] using polyethylenimine (PEI) transfecting reagent. The PEI stock solution was prepared by dissolving PEI (Sigma) in deionized water to reach 500 ng/μl. A total 4 μg of all four plasmid constructs including 1.2 μg of pMDLg/pRRE, 0.8 μg of pRSV_Rev, 0.4 μg of pMD2.G, and 1.6 μg of pLVX_GΔTM were diluted in 100 μl of 150 mM NaCl in a polypropylene tube. The PEI working solution (60 μg PEI per 1 μg DNA) was prepared by diluting 400 μl of the PEI stock in 350 μl of 150 mM NaCl. The PEI working solution was then transfer to the DNA tube. The DNA/PEI mixture was mixed gently and incubated at room temperature for 20–25 min. In the meantime, the culture medium was removed from the 10 cm^2^ plate containing LentiX-293T cells and gently replaced with 1 ml of OptiMEM (Gibco). Then, the DNA/PEI mixture was added into the culture plate in a drop wise manner and incubated at 37 °C with 5% CO_2_ for 5 h. After the incubation, the medium was removed and replaced with OptiMEM complete medium containing 10% FBS, 4% l-glutamine, and 1% streptomycin-ampicillin followed by further incubation for 48 h. The culture medium supernatant was then harvested and clarified by centrifugation at 2000 rpm for 10 min. The lentiviral titer was determined by quantitative RT-PCR (qRT-PCR).

### 2.2 Development of real time qRT-PCR for recombinant lentivirus detection

To generate GagP24 RNA transcript to be used as positive control for the recombinant lentivirus titration, GagP24 was amplified from pMDLg/pRRE plasmid [3] using the following primer pair: BamHI_Gagp24_F (ATATGGATCCCATATAGTATGGGCAAGC) and XhoI_Gagp24_R (ATATCTCGAGACTGTGTTTAGCATGGTG). The PCR product was cloned into pGEMT-easy vector (Promega) to construct a recombinant plasmid containing GagP24 sequence (pGagP24). The sequence and direction of GagP24 were verified by DNA sequencing. Subsequently, the pGagP24 construct was cut with *Bam*HI to linearize the plasmid which was the template to generate GagP24 RNA transcript by using T7 *in vitro* transcription kit (Promega) following the manufacturer instruction. The plasmid DNA was removed by incubation with 1 unit of DNaseI (Promega) at 37 °C for 30 min followed by incubation with StopDNaseI solution at 65 °C for 30 min. Then, ethanol precipitation was performed to concentrate the GagP24 RNA transcript [4]. GagP24 RNA copy number was calculated using an available online tool, NEBioCalculator™ (https://nebiocalculator.neb.com/#!/ssrnaamt). For standard curve optimization, 500 ng of the GagP24 RNA transcript was 10-fold serially diluted from 50 ng to 5 fg and used as the template for qRT-PCR. The real time RT-PCR was performed in triplicate using iTaq universal SYBR green one-step kit (Bio-Rad) and GagP24 specific primers: Gagp24_F (CTGTTAGAAACATCAGAAGGCTG) and Gagp24_R (CACACAATAGAGGGTTGCTACTG). The thermal cycling protocol consisted of reverse transcription step at 50 °C for 10 min, polymerase activation and DNA denaturation step at 95 °C for 1 min, followed by 35 cycles of denaturation at 95 °C for 10 s and annealing/extension at 60 °C for 15 s. Melt-curve analysis was also performed at 65–95 °C with 0.05 °C increment.

### 2.3 Determination of optimal concentration of puromycin

Puromycin titration was performed to optimize the minimum concentration that completely killed all cells within 3–5 days. Initially, 293T cells were seed at 2 × 10^5^ cells/well in a 24-well plate and incubated overnight at 37 °C with 5% CO_2_. On the next day, culture medium was removed from each well and then replaced with the culture medium containing puromycin (Sigma) at 0.5, 1, 3, 5, 8, 10, 25, 50, and 100 μg/ml, respectively. The medium was changed every 2–3 days and cell viability was examined every day under light microscope until all cells died.

### 2.4 Generation of a stable BEFV GΔTM secretory cell line

To generate stable cells expressing secretory GΔTM protein, 293T cells were transduced with the recombinant lentivirus. Initially, 293T cells were seeded at 1 × 10^5^ cells/well in a 6-well plate and incubated overnight. The recombinant lentivirus at multiplicity of infection of 5 was combined with OptiMEM medium containing 10 μg/ml polybrene (Sigma) before adding onto the 293T cells. At 48 h after the transduction, the culture supernatant was removed and replaced with a selecting culture medium containing 3 μg/ml puromycin (Sigma) to select stable protein expressing and antibiotic resistant cells. The selective culture medium was changed every 3 days until colonies of the stable protein expressing cells were observed. The puromycin survival cell clones were pooled for further clone isolation.

### 2.5 Isolation of monoclonal BEFV GΔTM secretory cell population

The pool of polyclonal stable cells that survived the puromycin selection was isolated by limiting dilution to obtain monoclonal stable cell lines. Briefly, the polyclonal stable cell pool was seeded into a T75 cm^2^ cell culture flask to reach 60% confluent and incubated overnight. On the next day, the spent culture medium was collected and further used as a conditioned medium to enhance the single cell growth. The stable cell pool was separated into individual cells by trypsin treatment and pipetting up and down. The cells were enumerated by using a hemocytometer. The cell suspension was then diluted in the conditioned medium to reach the final concentration of 5 cells/ml. One hundred microliters of the cell suspension was seeded into each well of a 96-well plate and incubated at 37 °C with 5% CO_2_. Eight days after incubation, wells containing a single colony were maintained while wells with more than a single colony were discarded. Each single colony was culture in the 96-well plate until confluent. Subsequently, the cells were culture in the selecting media while they were stepwise transferred to larger culture wells, e.g. from 48-, 24-, 12- and 6–well plates to culture flasks, respectively. During the stable cells were cultured in 24-well plate, each of the monoclonal colonies was examined for the expression of GΔTM protein by Western blot analysis as described previously [5,6].
